# Older women's well-being during the COVID-19 pandemic: individual, community, and contextual factors

**DOI:** 10.3389/fgwh.2024.1484469

**Published:** 2024-12-12

**Authors:** Andrew Banda, Jaco Hoffman, Vera Roos

**Affiliations:** ^1^ Optentia Research Unit, North-West University, Vanderbijlpark, South Africa; ^2^ Department of Demography, Population Sciences, Monitoring and Evaluation, University of Zambia, Lusaka, Zambia; ^3^ The Oxford Institute of Population Ageing, University of Oxford, Oxford, United Kingdom

**Keywords:** individual and community-contextual factors, older women, well-being, COVID-19, Zambia

## Abstract

**Objective:**

This article aims to examine the influence of individual and community-contextual factors on the well-being of older women in Zambia during the COVID-19 pandemic, drawing on Bronfenbrenner's process-person-context-time model.

**Methods:**

Secondary data from the nationally representative 2021 SEIA were used, and bivariate and logistic regression analyses were performed to determine factors associated with the well-being of older women during the COVID-19 pandemic.

**Results:**

Overall, 29% (613) of older women reported a decline in their well-being due to COVID-19. Older women in rural areas had lower odds of well-being [Adjusted Odds Ratio (AOR) 0.607, 95% 0455,0.809]. At the individual level, the well-being of older women during COVID-19 was associated with age (AOR O.362, 95% CI: 0.190,0.689) and being in paid work (AOR 0.737, 95% CI: 0.552,0.984). Despite education having a strong relationship with well-being, it had a weak effect on the well-being of older women during COVID-19. Community-level factors significantly associated with the well-being of older women amidst COVID-19 included attendance at public gatherings (e.g., church meetings, funerals) (AOR 1.465, 95% CI: 1.139,1.885) and perceived fear or anxiety due to COVID-19 (AOR 0.522, 95% CI: 0.392,0.696). A significant contextual-level factor was access to transport services during the pandemic (AOR 0.589, 95% CI: 0.390,0.890).

**Conclusion:**

COVID-19 has exposed the inadequacy of systems at different levels in meeting the needs of older women and promoting their well-being during emergencies. At the individual level, there is a need to support older women's livelihoods and educational opportunities. Despite limitations on social interactions during COVID-19, access to social gatherings and interactions supported older women's well-being. However, this was hampered by fear of contracting COVID-19 and the limitations in public transport that compromised their mobility to access services and visit people. A more extensive analysis of individual, community, and contextual factors should identify factors that support or compromise the well-being of older women during emergencies or shocks. There is a need for information about what livelihood strategies during and/or post shocks, or critical events such as COVID-19 could sustainably support older women's well-being.

## 1 Introduction

COVID-19 has emerged as one of the most significant social, economic, and public health challenges of the 21st century since World War II, affecting everyone globally but not equally (1, 2). Since December 2019, the SARS-CoV-2 virus has traversed every corner of the world, changing how people live, interact, socialise, work, and seek or receive services (3). As of June 2024, there have been more than 775,654,882 confirmed cases of COVID-19, including 7,051,876 deaths worldwide (World Health Organisation (4). The spread and effect of the pandemic appear to differ across continents, countries, regions and social groups. Most globally reported cases were from Europe and the American continents (4). In Africa, the reported numbers and proportions are relatively low, however, it is argued that the numbers in Africa likely underestimate the true magnitude of the pandemic due to low testing and detection capacity (5). In Zambia, with an estimated population of 19.6 million (Zambia Statistics Agency (6), over 343,135 confirmed cases have been reported with 4,057 deaths (Ministry of Health (7). Zambia generally has a young population with about 79% (15,570,950) of those under 35 years. However, the proportion of the population aged 50 years and over has steadily increased, averaging 8% (1,673,149) in 2022 and is projected to grow to about 10% in 2035 (8).

Recent evidence shows that the well-being of older people during COVID-19 was influenced by age, work status, living situations, access to health insurance, access to income, and the type of social network and support (9–14). According to the WHO (4), older people are at higher risk of not only experiencing severe illness from contracting COVID-19 but also bearing an excessively heavy burden due to other health, social and economic consequences (14). The impact of COVID-19 has been far-reaching, negatively affecting many sectors of Zambia's society ranging from the contraction of the economy, loss of employment and income, and in some cases loss of business (15). The pandemic revealed deep-rooted healthcare, psychological and socio-economic vulnerabilities that could be perpetuated due to gender inequalities (9, 16, 17). Mooi-Reci and Risman (18) argue that older people, particularly older women, bore the heaviest brunt of the pandemic because they were already a vulnerable section of society. This is further exacerbated by low educational attainment among older adults and high poverty estimated at 60%, with rural areas disproportionately affected at 78.8% relative to urban poverty (31.9%) (Zambia Statistics Agency (8). The interplay of these factors creates an environment where older people find it challenging to experience optimal well-being (19).

The COVID-19 pandemic has significantly altered the lives of older people across capabilities (livelihoods), aspects of well-being (subjective, material and relational), and the digital divide (20, 21). Livelihoods comprise capabilities, assets, and activities required for a means of living and when sustainable, it can cope with and recover from stresses and shocks (COVID-19 pandemic) (22). Livelihood assets specifically among older women include social interactions (social capital), capital assets (livestock, a business), financial support, access to natural resources and their physical well-being (19). The COVID-19 restrictions impacted these assets with business shutdowns, slow production processes, reduced farming activities, and job losses (23). It also led to a reduction in material provision in the form of food, money, and intangible social support and care (24).

Measures to avert the spread of the pandemic such as social distancing, lockdowns and stay-at-home diktats significantly changed how people interact and maintain social networks and connections (3). The reduced social interaction of older people and their networks threatened the social support they received, which is essential for their well-being (25). Particularly lockdowns and stay-at-home restrictions exacerbated loneliness and social isolation because it further reduced older persons already constrained social space (12). A push towards working from home and moving in-person interactions to digital platforms and technologies for social connection have seen significant growth among the general population. However, a study in the Americas found that older people fell on the wrong side of the digital divide, limiting their social interaction, and access to healthcare (26). Blomberg et al. (27) argue that particularly women were marginalised with limited access to technology and internet connectivity and therefore, had lower prospects impeding their ability to work, access services, and stay informed about the pandemic. Post-pandemic, older people continue to face challenges with technology and digital access that prevent the use and uptake of digital services due to deficient literacy or the general lack of ability to adapt to the use of digital services (10). They also argue that the tragedy of the digital divide during and post-pandemic is that older people were already previously disadvantaged both socially and, in their ability, to use digital services. The digital divide may exacerbate the already high levels of social isolation and loneliness even beyond the pandemic (10). In Zambia, online platforms such as Zoom and WhatsApp became essential in the provision of counselling services and conducting work and virtual social meetings, from which by far the majority of older persons were excluded participating in society (28).

The hedonic view of well-being was used in this study as it encompasses the thinking and feelings of an individual based on material or relational aspects in their context (29). Therefore, the research question guiding this article is what factors (individual, community or contextual) impacted older women's well-being during COVID-19.

To address the research question, Bronfenbrenner's process-person-context-time (PPCT) model is adopted. This model is useful as it emphasises the dynamic relationship between people, real-life events, and their physical and social environments (30, 31). The original bioecological theory considered contextual influences on human development and later included the integration of person, process and time variables (32, 33). The PPCT model explains the interplay of individual factors, and community-contextual factors within the temporal dimension; in this study related to the COVID-19 pandemic. In this article, the focus is on the functional relationship of the environment (social and physical) and older women as developing individuals. The temporal dimension of the PPCT model is relevant because COVID-19 accentuated already existing environmental deficiencies such as inadequate shelter, lack or inadequate medical facilities, poor sanitation, lack of electricity for cooking and lighting, insufficient food, and boredom due to lack of age-appropriate creative activities (34, 35). Pre-COVID-19, the environment already challenged older women in terms of disparities in access to food, healthcare, income and support services (36). COVID-19 further exacerbated affected older women's well-being to provide for their households, associated labour and care burdens, challenges to maintain relationships and social networks, and disruptions to social initiatives such as participation in membership clubs (20). The temporal dimension is relevant because of the protracted impact of post-pandemic consequences on older people who still spend more time at home and with fewer social networks (17), continuing to impact their well-being. Drawing on the 2021 SEIA survey data, this article addresses COVID-19 at the well-being nexus.

## 2 Methods and data

### 2.1 Data source

The SEIA is a multi-faceted survey that includes the Covid-19 Impact Assessment, the Survey of Well-being via Instant Frequent Tracking (SWIFT), and the Cross-sectional Living Conditions Monitoring Survey. The survey covered key topics relating to knowledge, attitudes and practices, the socioeconomic effects of COVID-19 on households, COVID-19 vaccine awareness and willingness to be vaccinated, as well as access to healthcare services. The SEIA is a nationally representative cross-sectional population-based household survey that estimates national, provincial and residence (rural/urban) levels. The survey employed a two-stage stratified sampling cluster sample design: In the first stage, 420 enumeration areas (EAs) were selected with probability proportional to the size (PPS) of the stratum. Each EA comprised the number of households enumerated in the 2010 census of population and housing. A listing of all the households in each selected EA was conducted to generate an updated number of households.

**Figure 1:**
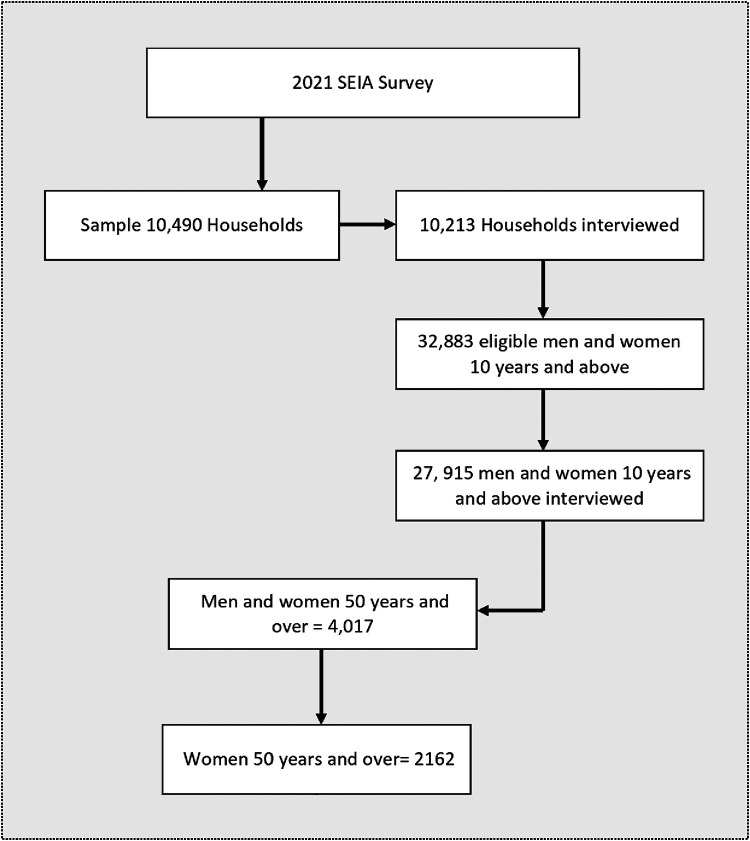
Sample derivation of older women 50 years and older, SEIA 2021.

In the second stage, systematic sampling was employed to select 25 households from each EA. In situations where the listed households in an EA were 25 or fewer, all households in such EAs were interviewed. A total sample of 10,490 households were sampled and 10,213 households were interviewed, representing a response rate of 97%. Of all the interviewed households, 32,883 men and women aged ten (10) years and older were identified; of the total eligible individuals for interviews, 27,915 interviews were conducted. For this study, the sample size was limited to older women aged 50 years and over, residing in rural and urban areas. Figure 1 shows the sample size determination of the study.

### 2.2 Study variables and measurement

#### 2.2.1 Dependent variable

The 2021 SEIA measured the socioeconomic effect of COVID-19 by asking respondents what the main effect of COVID-19 on their lives was. A 9-response category self-assessed question was used. The study also asked respondents how often they felt depressed, nervous, and could not control worrying; a 4-response category Linkert scale (all the time, often, sometimes and not at all) was used to assess the frequency. Based on these questions, an outcome variable “well-being” was constructed.

The two sets of questions were used to compute a proxy outcome variable of well-being. Well-being is traditionally measured using standard instruments of subjective well-being (37). However, the self-assessed socioeconomic effect of COVID-19 and experiences of depression, nervousness, and inability to control worrying provide a holistic picture of older people's well-being and cover two important aspects of the socioeconomic effects and psychological experiences of older people, which can be considered a good measure of older persons’ well-being.

The “well-being” outcome variable was computed into a discrete binary variable: coded as (1) if the respondent reported that they have had experienced depression and nervousness and could not control worrying. Those who reported that COVID-19 had no effect, that they did not experience depression or nervousness or could control worrying were coded as (0).

Further, responses relating to whether an older person had lost a job, lost savings, could not pay for a loan or mortgage, could not afford to buy food, had to close their business, or had their income and/or production reduced, were computed as the economic impact of COVID-19.

#### 2.2.2 Independent variable

Independent variables were broadly categorised into four: demographic and socio-economic characteristics; older people's perception of COVID-19; health and access to healthcare; and variables relating to social interaction during the pandemic.

#### 2.2.3 Demographic and socioeconomic characteristics

Variables considered for older people included age in years (5-year intervals) coded as 1 = 50-54, 2 = 55-59, 3 = 60-64, 4 = 65-69, 5 = 70-74,6 = 75-79, 7 = 80-84, and 8 = 85 + . Marital status was coded into 4 distinct groups; 1 = never married, 2 = married/cohabiting, 3 = divorced/separated, and 4 = widowed. Residence was coded in two, 0 = Urban and 1 = Rural. Regarding education, the respondents were asked whether they ever attended school instead of indicating their highest level of education as there were many cases in which the highest level of education attained was unknown, coded as (1) if ever attended school and (0) if otherwise. Work (paid work) is essential to older peoples’ well-being; respondents were thus asked if they were in any paid work or unpaid family business, this was coded as (1) if they were in paid work and (2) if otherwise.

#### 2.2.4 Older people's perceptions of COVID-19

Because COVID-19 is a novel pandemic, determining older women's perception of the seriousness of the virus and the risk of infection is essential to get an understanding of its impact on well-being. Further, respondents were asked if they had ever heard of anyone discriminated against because of COVID-19 and whether they would discriminate against someone with COVID-19.

#### 2.2.5 Health and access to healthcare services

Two questions were used to assess health and access to healthcare. Respondents were asked if they had a medical condition (diabetes, hypertension, cancer, renal kidney disease, chronic pulmonary (lung) disease, tuberculosis, arthritis, or HIV. Respondents were asked to respond (yes) if they had a condition and (no) if they reported otherwise. Based on these conditions, an index variable ‘ill health’ was generated, coded as (1) if they reported having any conditions and (0) if otherwise.

Regarding access to treatment, respondents were asked if, in the last 12 months (since March 2020), they needed treatment but could not access healthcare. This variable was coded as (0) if yes, needed treatment but did not get it and (1) if they did seek treatment and received it.

#### 2.2.6 Social interaction during COVID-19 and well-being

To measure the extent of social exclusion of older women from community activities, respondents were asked if they had attended any event (church, funeral, parties, work meetings/workshop, bar/restaurant) since March 2020 (12 months before the survey). The variable attendance to public gatherings is divided into five distinct categories; 1 = Did not attend any event, 2 = Attended a church meeting, 3 = Bar/restaurant/parties, 4 = Work meetings/workshop, and 5 = attended a funeral. Similarly, respondents were asked if they had been on a public bus the last seven days before the survey coded as (1) if they used public transport and (2) if otherwise.

### 2.3 Statistical analysis

The statistical analyses were done using STATA version 14. Three staged analyses were carried out. The first step involved a univariate analysis to describe all the variables. Secondly, a bivariate analysis with chi-square statistics was performed to test the independence of distribution between the independent variable and the outcome variable. The third stage entailed that logistic regression was performed to assess the net association of the independent variables with the outcome variable. Three models were generated: (1) a model with older women's characteristics only, (2) a model with older women's perceptions about COVID-19, and (3) a model with all the variables. Only significant variables from the bivariate analysis using Pearson's chi-square test (*P* < 0.05) (5%) were added to the final model (Model 3).

### 2.4 Ethical approval

Permission to use the dataset was sought from the Zambia Statistical Agency (ZamStats). Ethics approval was granted from the North-West University Human Social Sciences Research Ethics Committee for secondary analysis of the SEIA data under ethics number NWU-01152-22-A7.

## 3 Results

The results are presented at different levels; univariate and bivariate analysis to understand the description and relationship between and among variables. The last part describes the causal relationship of explanatory variables regarding the well-being of older women in Zambia during the COVID-19 pandemic.

### 3.1 Participants characteristics

Table 1 describes the participants’ demographic, socioeconomic, social relational, and health characteristics. There were 2,162 older women 50 years and older in both rural and urban settings that constituted the sample, accounting for 54% of the total number of older people in the sample. The mean age of participants in the sample was 62.1 years [Standard Deviation (SD) = 9.6]. More than half (60.1%) of respondents were residing in rural areas. A majority (83%) of older women reported ever attending school. Regarding work status, 52.7% of respondents were in some form of paid work. A majority (72.6%) of participants perceived COVID-19 to be a very serious problem of the moment (2021-2022 survey period) while only 6.7% indicated that they have heard of someone being discriminated against on account of COVID-19 and 11.0% of respondents reported that they would not discriminate against someone because of COVID-19. Slightly over half of respondents had attended a public gathering (church), 14.2% had used public transport in the two weeks before the survey and 26.2% owned a bicycle as a means of transport. About 55% of respondents were economically impacted by the pandemic and less than one-third (29.2%) had had access to a social protection intervention during the pandemic.

**Table 1:** Participants’ characteristics.

Variables		Women (*N* = 2,165)	
		*f*	*%*
Age (grouped)	M(SD)	62.1 (9.6)	
	50–54	557	26.0
	55–59	459	22.2
	60–64	389	17.8
	65–69	279	22.6
	70–74	197	8.7
	75–79	145	6.5
	80–84	78	3.6
	85+	58	2.5
Marital status	Never married	59	2.5
	Married/cohabiting	1,057	49.6
	Divorced/separated	249	11.3
	Widowed	797	36.6
Ever attended school	Yes	1,778	83.4
	No	384	16.4
Residence	Rural	1,318	60.1
	Urban	844	39.9
Work status (Paid work)	Yes	1,151	52.7
	No	1,011	47.3
Perception about seriousness of (COVID-19) ^a^	Serious	1,440	72.6
	Neutral	200	9.7
	Not serious	373	17.8
Heard of older people with COVID-19 discriminated ^a^	Yes	149	7.1
	No	1,864	92.9
Would you discriminate someone with COVID-19 ^a^	Yes	231	11.0
	No	1,782	89.0
Attendance (public gathering)	Funeral	223	10.1
	Church	1,136	51.9
	Bar/restaurant	14	0.7
	Work meeting/workshop	43	2.1
	Did not attend any event	746	35.2
	No	1,898	85.8
Would you seek healthcare if you had fever? ^a^	Yes	1,841	92.3
	No	94	4.1
	Not Sure	78	3.6
Unmet need for medical care (since March, 2020) ^b^	Yes	251	11.0
	No	1,807	84.0
Economically impacted by COVID-19	Yes	1,186	54.7
	No	976	45.3
Did you receive any social protection package	Yes (SCT, emergency cash transfer, FSP)	675	29.2
	No	1,487	70.8
Use of public transport last 7 days	Yes	264	14.2
	No	1,898	85.8
Ownership of means of transport ^c^	Bicycle	576	65.2
	Car	93	12.8
	Motorcycle	29	4.0
	Scotch cart	93	11.4
	Canoe	68	6.5

M, mean; SD, standard deviation.

^a^ 149 (5.6%) missing cases.

^b^ 104 (5.0%) missing cases.

^c^ 1,302 (59.8%) missing cases.

Table 2 shows the relationship between the well-being of older women and selected individual, community, and contextual characteristics of respondents. Analysis was conducted by establishing the relationship between older women's perceived well-being and their demographic, social, economic and health factors. Significant differences in the well-being of older women during the pandemic were found in the place of residence, age group of participants, marital status, and level of education. There were also significant differences in well-being among older women's perceptions of the seriousness of COVID-19, whether they have heard about someone being discriminated against or would discriminate against anyone because of COVID-19. A significant relationship was observed between access to social protection interventions during the pandemic and the well-being of older women.

**Table 2:** Relationship between older women's well-being and selected characteristics, SEIA 2021.

Variables		Well-being (Older women 50+) (*N* = 2,162)				*P*-Value
		Yes	95% CI	No	95% CI	
Residence	Urban	78.9	[75.5, 82.0]	21.1	[18.0, 24.5]	
	Rural	66	[63.1, 68.8]	34	[31.2, 36.9]	0.000***
Age group	50–54	74.6	[70.3, 78.5]	25.4	[21.5, 29.7]	
	55–59	72.4	[67.6, 76.8]	27.5	[23.2, 32.3]	
	60–64	75	[69.9, 79.5]	25	[20.6, 30.1]	
	65–69	68.6	[62.2, 74.4]	31.4	[25.6, 37.8]	0.000***
	70–74	69.5	[61.8, 76.2]	30.5	[23.8, 38.2]	
	75–79	72.4	[63.3, 79.9]	27.6	[20.1, 36.7]	
	80–84	48.6	[36.2, 61.1]	51.4	[38.9, 63.8]	
	85+	43.4	[30.1, 57.8]	56.6	[42.2, 69.9]	
Marital status	Never married	73.8	[57.8, 85.3]	26.2	[14.7, 42.2]	
	Married/cohabiting	70.6	[67.3, 73.8]	29.4	[26.2, 32.7]	0.051*
	Divorced/separated	63.2	[55.8, 70.0]	36.8	[30.0, 44.2]	
	Widowed	73.4	[70.0, 76.5]	26.6	[23.5, 29.9]	
Ever attended school	Yes	72	[69.5, 74.3]	28	[25.7, 30.5]	
	No	67	[61.6, 72.1]	33	[27.9, 38.4]	0.000***
Perception about seriousness of COVID-19	Very serious	76.3	[73.8, 78.8]	23.6	[21.2, 26.2]	
	Neutral	66.9	[59.2, 73.8]	33.1	[26.2, 40.8]	0.000***
	Not serious	60.2	[54.3, 65.7]	39.8	[34.3, 45.6]	
Heard of older person with COVID-19 discriminated	Yes	82.4	[74.2, 88.4]	17.6	[11.6, 25.8]	
	No	71.8	[69.4, 74.1]	28.2	[25.9, 30.6]	0.000***
Would discriminate someone with COVID-19	Yes	69.9	[62.6, 76.3]	30.1	[23.7, 37.4]	
	No	72.9	[70.5, 75.2]	27.1	[24.8, 29.5]	0.000***
Attendance (public gathering)	Did not attend any event	68	[64.1, 71.6]	32	[28.4, 35.9]	
	Funeral	72.1	[65.0, 92.4]	27.9	[21.8, 34.9]	
	Church	72.5	[69.5, 75.3]	27.5	[24.7, 30.5]	0.175
	Bar/restaurant/parties	85.1	[59.5, 95.7]	14.9	[4.2, 40.5]	
	Work meeting/workshop	81.3	[61.1, 92.4]	18.7	[7.8, 38.9]	
Use of public transport last 7 days	Yes	76.4	[64.9, 84.9]	23.6	[15.1, 35.0]	
	No	65.4	[62.4, 68.2]	34.6	[31.8, 37.6]	0.042*
Would you seek medical care if you felt unwell?	Yes	68	[64.8, 70.9]	32	[29.0, 35.2]	
	No	74.6	[60.7, 84.9]	25.3	[15.1, 39.3]	0.000***
	Not sure	52.9	[38.4, 55.6]	47.1	[33.5, 61.1]	
Received social protection	Yes	69.7	[65.1, 73.9]	30.3	[26.0, 34.9]	
	No	63.7	[59.7, 67.3]	36.3	[32.7, 40.0]	0.044*
Total		1,549		613		

* *p* < 0.1.

** *p* < 0.05.

.*** *p* < 0.01.

Table 3 shows a summary of Cronbach's alpha, the results show an alpha of 0.34. implying that the variables have a weak conceptual relationship, However, this could be because of the heterogeneity of constructs being measured to explain the well-being of older women (individual and community-contextual factors). As such the variables included are meant to explore related but distinct aspects of the well-being of older women during COVID-19. Further, the study was conducted at the height of the COVID-19 pandemic which might have influenced the variability in responses rather than the lack of reliability.

**Table 3:** Cronbach Alpha test.

Item	Observation	Sign	Item-test correlation	Item-rest correlation	Average interitem correlation	Alpha
Well-being	2,497	-	0.377	0.127	0.039	0.290
Age group	2,497	+	0.485	0.250	0.029	0.230
Marital status	2,497	+	0.394	0.148	0.037	0.275
Ever attended school	2,497	+	0.454	0.211	0.032	0.251
Work status (Paid Work)	2,497	+	0.417	0.174	0.036	0.270
Perceived seriousness of COVID-19	2,320	+	0.379	0.128	0.039	0.286
Heard of an older person with COVID-19 discriminated	2,320	+	0.226	-0.034	0.051	0.351
Would you discriminate someone with COVID-19	2,497	-	0.232	−0.028	0.051	0.349
Attendance (public gathering)	2,497	-	0.325	0.079	0.042	0.306
Use of public transport (Last 7 days)	2,497	+	0.357	0.114	0.039	0.291
Would you seek medical care (signs of COVID)	2,320	+	0.313	0.056	0.044	0.316
Total Scale					0.040	0.314

### 3.2 Factors associated with the well-being of older women during COVID-19

Weighted logistic regression analysis on factors related to the well-being of older women during COVID-19 is shown in Table 3. Results show that factors associated with the well-being of older women during the COVID-19 pandemic are residence, age, being in paid work, perception of the seriousness of the pandemic, attendance of public gatherings, and the ability to use public transport during the pandemic.

#### 3.2.1 Individual characteristics of older women and well-being during COVID-19

Table 4 shows that the odds of well-being were lowest among older women residing in rural areas and those in advanced age. The odds of well-being among older people in rural areas were 34.4% lower compared to older women in urban areas [Adjusted Odds Ratio (AOR) 0.656, 95% CI;0.507,0.849] and 63.8% older women in the age group 80-84 were less likely to report better well-being compared to older women aged 50-54 years (AOR O.362, 95% CI: 0.190,0.689). In terms of older women's involvement in paid work, older women who were not in paid work were 26.3% less likely to have better well-being compared to older women in paid work (AOR 0.737, 95% CI: 0.552,0.984). Although coefficients for older women's marital status and ever-attended school (education) were not statistically significant in the regression analysis, there is a strong relationship between education, marital status and the well-being of older women during COVID-19 (Table 2).

**Table 4:** Logistic regression of factors associated with the well-being of older women during the COVID-19 pandemic, SEIA 2021.

Dependent variable: well-being of older women (50 + years)								
Characteristics	Model 0		Model I		Model II		Model III	
	OR	95% CI	OR	95% CI	OR	95% CI	OR	95% CI
Residence of respondent								
Urban (RC)			1	[1,1]			1	[1,1]
Rural			0.538***	[0.425,0.681]			0.656**	[0.507,0.849]
Age group								
50–54 (RC)			1	[1,1]			1	[1,1]
55–59			0.848	[0.613,1.171]			0.937	[0.667,1.316]
60–64			0.934	[0.662,1.319]			1.07	[0.743,1.541]
65–69			0.696*	[0.478,0.013]			0.788	[0.531,1.171]
70–74			0.743	[0.489,1.130]			0.795	[0.499,1.266]
75–79			0.867	[0.522,1.439]			0.956	[0.535,1.706]
80–84			0.328***	[0.181,0.593]			0.362**	[0.190,0.689]
85+			0.296***	[0.155,0.567]			0.311**	[0.146,0.660]
Marital status								
Never married (RC)			1	[1,1]			1	[1,1]
Married/cohabiting			0.864	[0.423,1.768]			0.889	[0.429,1.845]
Divorced/separated			0.756	[0.352,1.624]			0.733	[0.335,1.606]
Widowed			1.233	[0.599,2.538]			1.151	[0.551,2.402]
Ever attended school								
No (RC)			1	[1,1]			1	[1,1]
Yes			0.923	[0.692,1.231]			1.172	[0.837,1.640]
Work status (paid work)								
Yes (RC)			1	[1,1]			1	[1,1]
No			0.889	[0.709,1.114]			0.737**	[0.552,0.984]
Perception about seriousness of COVID-19								
Yes, serious (RC)					1	[1,1]	1	[1,1]
Indifferent					0.667*	[0.465,0.956]	0.687*	[0.477,0.989]
Not serious					0.493***	[0.373,0.653]	0.522***	[0.392,0.696]
Heard of older person with COVID-19 discriminated								
Yes (RC)					1	[1,1]	1	[1,1]
No					0.586+	[0.343,1.002]	0.639	[0.369,1.107]
Would you discriminate someone with COVID-19								
Yes (RC)					1	[1,1]	1	[1,1]
No					1.198	[0.837,1.714]	1.131	[0.796,1.609]
Attendance (public gathering)								
Did not attend any event (RC)					1	[1,1]	1	[1,1]
Church					1.391**	[1.086,1.783]	1.465**	[1.139,1.885]
Bar/restaurant/parties					1.817	[0.441,7.491]	1.76	[0.420,7.380]
Work meeting/workshop					1.77	[0.623,5.025]	1.626	[0.547,4.830]
Funeral					1.059	[0.723,1.551]	1.044	[0.708,1.539]
Use of public transport last 7 days								
Yes (RC)					1	[1,1]	1	[1,1]
No					0.474***	[0.320,0.704]	0.589*	[0.390,0.890]
Would you seek medical care if you had a fever?								
Yes (RC)					1	[1,1]	1	[1,1]
No					1.303	[0.746,2.277]	1.432	[0.797,2.573]
Not sure					0.469**	[0.278,0.790]	0.498*	[0.287,0.863]
Intercept	2.465 ***	[2.219,2.737]	5.019***	[2.354,10.696]	7.291***	[3.604,14.751]	10.687***	[4.317, 26.453]
N (Older women 50+)	2,162		2,162		2,013		2,013	
R squared (%)	0		3.6		3.7		5.9	

Exponentiated coefficients; 95% confidence intervals in brackets.

* *p* *<* *0.1.*

** *p* *<* *0.05.*

^***^ *p* < 0.01.

#### 3.2.2 Socio-economic effects of COVID-19 on older women

**Figure 2:**
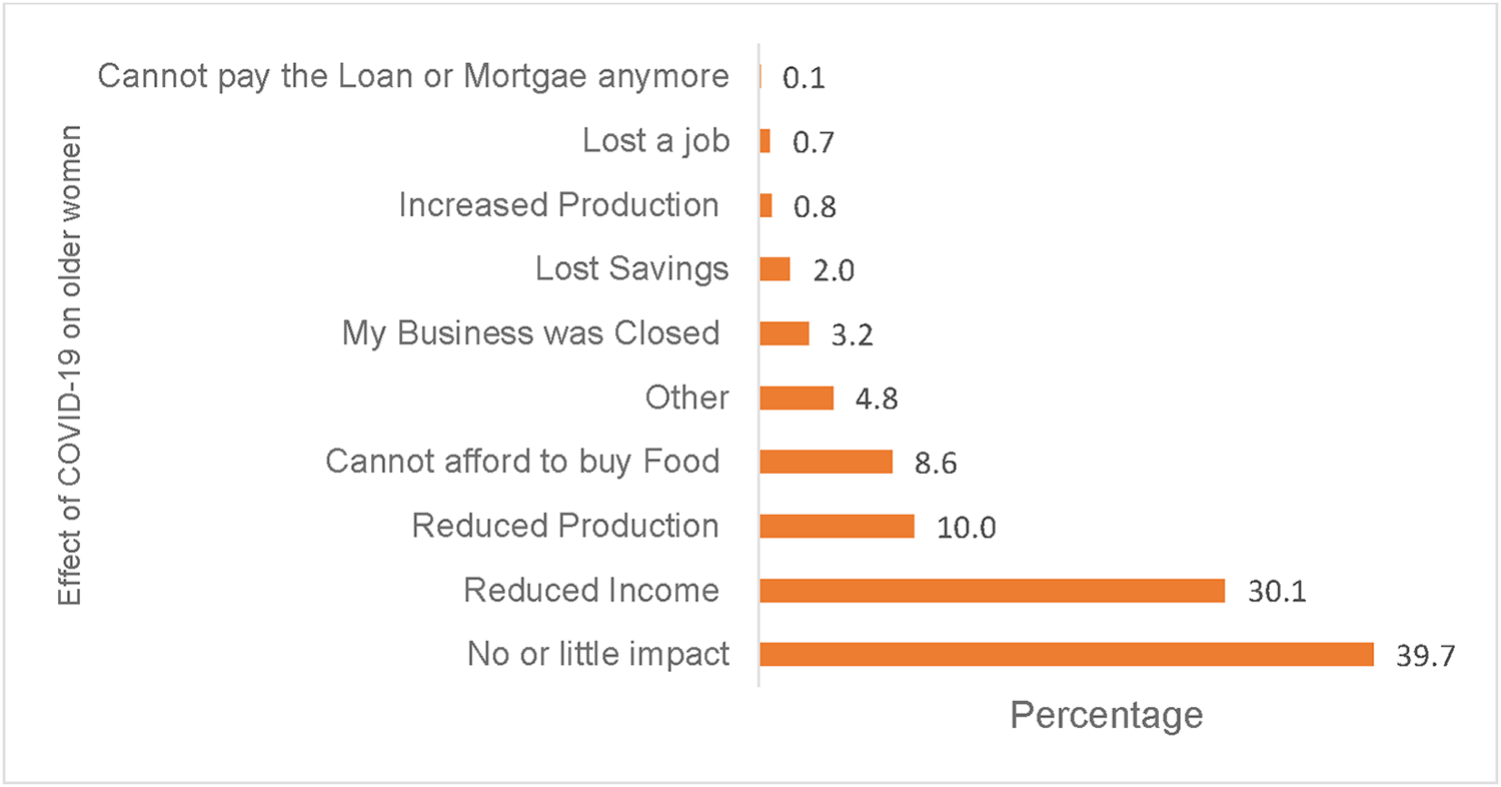
Effects of COVID-19 on older women's livelihood, SEIA 2021.

The impact of the pandemic was assessed by asking older people what aspects of their livelihood had changed because of COVID-19 pandemic measures. Figure 2 shows the effects of the COVID-19 pandemic as reported by older women. The results show that close to one-third (30.1%) of older women reported a reduction in income, 10% reported a reduction in agriculture productivity, 3% had their businesses closed, and 2% lost savings during the COVID-19 pandemic (Figure 2). About 40% of older women reported little or no impact associated with COVID-19. The subsequent prevailing question, therefore, is whether COVID-19 had no impact on the well-being of some older women or what obscured/mitigated the impact of the pandemic on their well-being.

#### 3.2.3 Perceived seriousness of the impact of COVID-19 and discrimination

COVID-19 was associated with great fear, stigma, and anxiety, particularly among older people who had a disproportionately high risk of infection and severe illness. Older people were asked how they perceived the seriousness of the problem of the COVID-19 pandemic on people's general well-being. Table 3 shows that older women who perceived the COVID-19 pandemic as a serious problem had 47.8% lower odds of well-being compared with older women who perceived the COVID-19 pandemic not to be a serious problem (AOR 0.522, 95% CI: 0.392,0.696). Among older women who were indifferent about the seriousness of the pandemic, their odds of well-being were 31.1% lower compared to older women who reported it as a serious problem (AOR 0.687, 95% CI: 0.477, 0.989). The coefficients on discrimination against older women with COVID-19 failed the significance test.

#### 3.2.4 Diminishing social interaction (attendance of public gatherings)

Social interaction and networks in older ages are essential aspects that support their well-being. However, COVID-19 restricted many aspects of daily living for older people and the general population. Results show a significant relationship between the well-being of older women and attendance at public events such as funerals, church gatherings, socializing at the tavern or work meetings; similarly, older women who reported stepping out of their households to take a bus or using the public transport system appear to have better well-being (*p* < 0.05) (Table 2).

Table 3 shows that attendance of community public gatherings such as church services during COVID-19 increased the odds of well-being among older women. The results show that the odds of well-being were 46.5% higher among older women who attended a church service compared to older women who had not attended any event (AOR 1.465, 95% CI 1.139,1.885). On the other hand, older women who reported not using public transport in the last seven days before the survey had 41.1% lower odds of well-being than older women who had used public transport (AOR 0.589, 95% CI: 0.390,0.890).

#### 3.2.5 Ownership of household assets, adaptation and coping strategies

**Figure 3:**
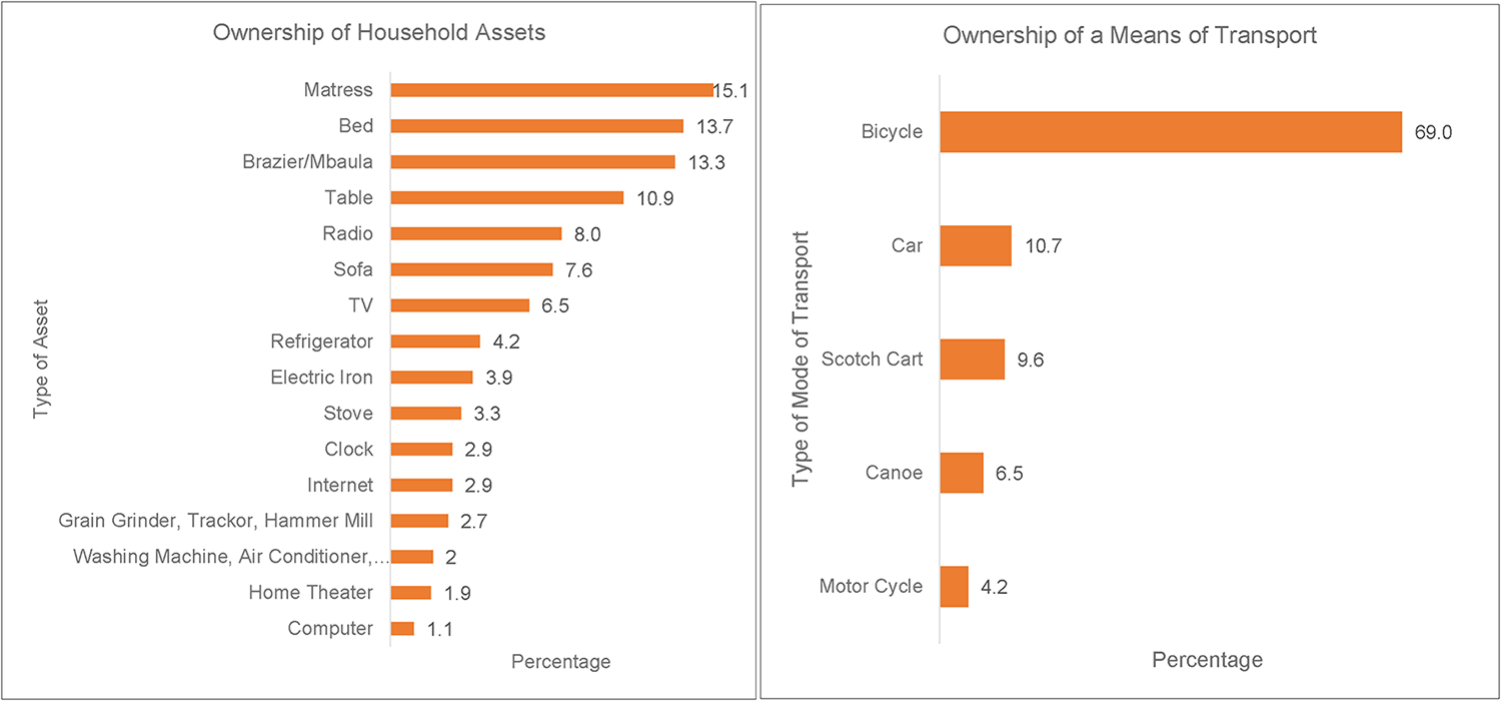
Ownership of household assets and means of transport.

Results show that COVID-19 diminished social interactions among older women, negatively affecting their socio-economic well-being. Figure 3 shows the ownership of household assets and ownership of a means of transport among older women. The most common assets owned by older women included mattresses (15.1%) and beds (13.7%) for sleeping, braziers (13.3%) for cooking, and radio (8%) and television sets (6.6%) for accessing information. The most common modality of transport owned by older women was bicycles (69%).

**Figure 4:**
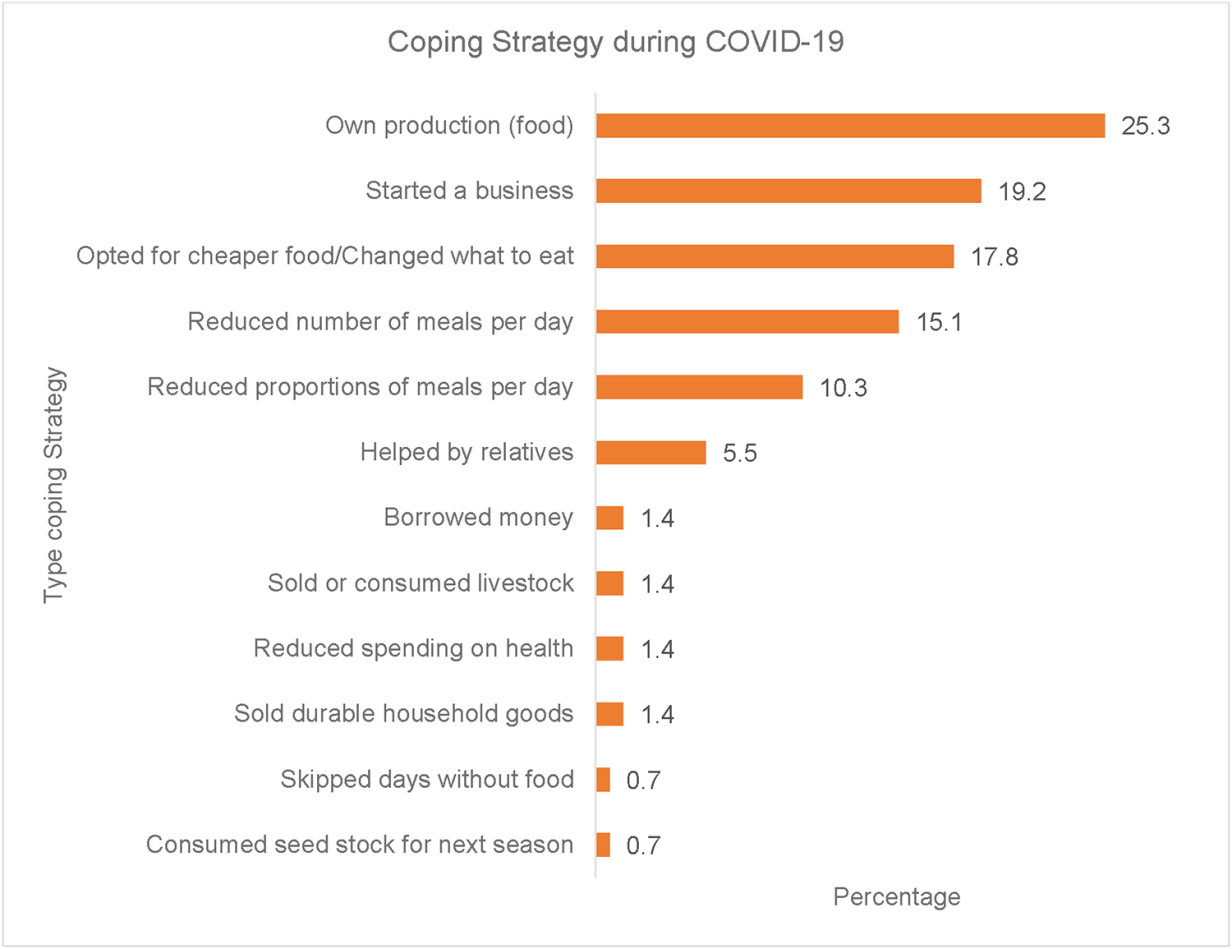
Coping strategies adopted by older women during COVID-19.

Regarding coping strategies, common coping strategies among older women included having their source of food (own production (25.3%), while other older women (19.2%) started a business: often petty trading of small food-related merchandise such as tomatoes and onions. Meanwhile, older women reported a change or reorganisation in what or how to eat, with 17.8% opting for cheaper foodstuff whereas 15.1% of older women reduced the number of meals per day and 10.3% reduced food proportions per day (Figure 4).

## 4 Discussion

This study examined the individual, community, and contextual factors during COVID-19 associated with the well-being of older women in Zambia using secondary data from the 2021 SEIA survey. Bivariate and multivariate regression analyses were used. Drawing on the PPCT model, age and work status as *individual-level factors* significantly negatively affected women's well-being. Older women in advanced ages reported a lower likelihood of well-being compared to younger counterparts in their 50s and 60s, possibly because according to literature (20, 38), COVID-19 exacerbated the already challenging physical environment for older women related to access of healthcare, food, and income. Concerning work status, the results show that older women who were not in paid work had lower odds of well-being. On the other hand, the results show a surprising feature where 40% of older women reported that COVID-19 had no or little impact on general well-being (Figure 2). Several reasons could explain this finding. Henning-Smith (39) argues that low-resource settings in a developing country like Zambia are already poorly resourced in terms of healthcare and other basic needs, thus, despite the impact of COVID-19, older women may have already experienced the effects of precariousness or are accustomed to living within the constraints of the limited resources available within their settings. However, the question remains: Are older women in Zambia insulated from or resilient in coping with external shocks or are older women more consumed with their daily basic needs for survival? Further, the results confirm a narrative among older people in Debre Markos Town, Ethiopia who argued that “Hunger would kill us instead of COVID-19” and considered the practice of social distancing to fight COVID-19 as an unwelcome luxury for people whose livelihood depends on begging and petty trade (40).

Profoundly, the findings from the study by Takele et al. (40) uncover deeper reflection on access to means of survival such as food, shelter, and how to provide care to others. Older women generally, may have been more concerned about sustaining their daily needs than worrying about the potential threat of COVID-19. Greteman et al. (41) observe in their study on rural and urban differences in perception, behaviours and healthcare disruption during COVID-19 that rural people were less concerned about COVID-19 within their communities. According to the United Nations World Economic Forum, the COVID-19 fallout may be worse for women than men (42). Women are being squeezed out of production and market circuits, quickly losing livelihood strategies and getting relegated to unpaid and invisible household work, or absorbing additional caregiving responsibilities on top of their unpaid household and care work (20, 43). Working-from-home schedules exacerbated the pressure on working women as the division of labour in the home remains highly gendered in most developing countries (43), especially in patriarchal societies like Zambia. However, the gendered dimension highlighted in this paper cautiously suggests that women may generally be resilient to shocks despite the disproportionate limitations they are subjected to (44). The results are consistent with Emerson et al. (11) who argued that more women reported being engaged in healthy coping behaviours than men and that more women reported adopting survival strategies such as using phones to communicate with others.

The positive, although not significant relationship between the well-being and education of older women, could be explained by the fact that COVID-19 took the world by surprise due to its life-threatening consequences and the subsequent extreme measures implemented (e.g., global lockdowns and social distancing). It is argued that this perceived threat posed by the COVID-19 pandemic triggered people's autonomic nervous systems irrespective of their educational levels (see 45, 46). Fear, stress, and uncertainty due to a real threat, activate autonomic nervous system dysregulation (45). It is within this threatening COVID-19 context, that older Zambian women expressed the importance of community gatherings. Literature confirms that meaningful people connections support co-regulation and ultimately, well-being because in a “state of connection, health, growth, and restoration are possible” (46). However, visiting friends was challenging because of the restrictions and lockdowns, and these interventions directly impacted older women (47). The temporal dimension of the PPCT model emphasises the prolonged consequences of disrupted social connections for older individuals’ well-being.

Considering *community-contextual level factors*, access to healthcare during the peak of the pandemic has become even more difficult than usual among older people (48). Simfukwe et al. (49) observed, that low-resource settings often rely on informal or public transport to move to points of service. The plight of older people, particularly women have been exacerbated by long-distances, poor service delivery, and in some areas with services such as healthcare are inadequate and non-existent with their material well-being, relationships, and social networks being significantly further reduced (50); HelpAge, 2021 (20);.

Diminishing social interaction exacerbated by restrictions to control the spread of the pandemic implied that older women lost their livelihoods, often earned through petty trading. To mitigate this situation, most households relied on their household assets to cope with the impact of COVID-19 (20). The study demonstrates the centrality of household assets such as basic household goods; beds, mattresses for sleeping, the radio and TV for entertainment, and ownership of a mode of transport such as bicycles to facilitate movement to essential services (51).

The findings of this article foreground the need to tailor interventions that can influence individual, community, and contextual issues that ultimately affect older women's well-being, particularly in times of shock. According to Morgan (52), the disruption caused by COVID-19 calls for a redefined focus not only on socio-demographic and health factors or provision of health services in addressing the impact of COVID-19 but also emphasises the centrality of social, economic and community inherent measures to mitigate the impact of COVID-19. The study also demonstrates that there is a definite need for action, both in the short and long term, to improve the provision of services such as economic opportunities, healthcare, food and other social services among older women to provide a safety net in times of unpredicted crises like pandemics and other related natural disasters.

Programmes related to the well-being of older women should adopt a multi-pronged approach and involve strategies such as availing credit and financial services and promoting market access and to support the development of a sense of community and social protection (13). Through conversion, these resources might enhance older people's capabilities to adapt to changing circumstances and the effects of the COVID-19 pandemic during and beyond shocks (53). Additionally, the quest for access to social support services as well as mental health services for older women are critical for the well-being of older women (24). It is also important to promote overall well-being through access to lifelong learning opportunities and skills training targeted at women. Although the particularity of COVID-19 as a perceived threat might have prompted an autonomic nervous system dysregulation [see (45, 46)], a study on the impact of education attainment on older people's well-being found that each additional year of education attainment improved the well-being of older persons, and education attainment over the life course is a paramount driver for many social, economic and health outcomes (19, 54). Life course learning or education is a crucial driver of well-being as it facilitates access to services, enhances choices, and leads to the possibility for people to live a flourishing life (55, 56). This underscores a life course approach to education for future generations of older women in terms of their capabilities and skills to negotiate pandemics/disasters (57).

The COVID-19 pandemic is a foretoken for developing countries to strengthen the livelihoods of older women regardless of setting, through deliberate interventions that safeguard these already marginalised and poorly resourced groups. Deliberate actions should be taken to enhance access to technology and implement digital literacy among older women to mitigate future eventualities of a similar scale as the COVID-19 pandemic.

When interpreting the results certain limitations should be considered: The survey data obtained the general socio-economic impact of COVID-19 on the household and could have missed salient aspects unique to older women in times of distress (pandemics). Complementing the survey data with qualitative interview data might have yielded nuanced insights to be considered for future research on the well-being of older women during and after disasters and shocks. Nevertheless, the study makes a compelling argument for focusing on a marginalised group of people (older women) in environments not conducive to their development.

## 5 Conclusion

The article juxtaposed the disruptive impact of COVID-19 and older women's well-being on the individual and community, and contextual levels. Unpredictably, the well-being of older women in rural Zambia did not significantly change for the worse, despite the severe health and social well-being implications that accompanied the COVID-19 pandemic. Whether the well-being of older women in deprived contexts was less optimal before the start of the pandemic because they survive with what they have, calls for a further critical and in-depth analysis. The tenacity of older women should, however, not be in question, as they have demonstrated their ability to overcome and persevere challenges in the face of adversity. This article draws attention to several systemic lags in managing pandemics such as COVID-19 by applying a blanket approach, impacting already vulnerable groups of people, and situating individuals to manage and cope with the implications.

## Data Availability

The original contributions presented in the study are included in the article/Supplementary Material, further inquiries can be directed to the corresponding author.
